# Long noncoding RNA signatures in intrauterine infection/inflammation-induced lung injury: an integrative bioinformatics study

**DOI:** 10.1186/s12890-023-02505-5

**Published:** 2023-06-06

**Authors:** Jiarong Pan, Canyang Zhan, Tianming Yuan, Weizhong Gu, Weiyan Wang, Yi Sun, Lihua Chen

**Affiliations:** 1 Department of Neonatology, Children’s Hospital, Zhejiang University School of Medicine, National Clinical Research Center for Child Health, Hangzhou, 310052 Zhejiang China; 2 Zhejiang Key Laboratory for Diagnosis and Therapy of Neonatal Diseases, Children’s Hospital, Zhejiang University School of Medicine, National Clinical Research Center for Child Health, Hangzhou, 310052 Zhejiang China

**Keywords:** Intrauterine infection, Inflammation, Lung injury, Long noncoding RNA

## Abstract

**Background:**

Intrauterine infection/inflammation can result in fetal and neonatal lung injury. However, the biological mechanisms of intrauterine infection/inflammation on fetal and neonatal lung injury and development are poorly known. To date, there are no reliable biomarkers for improving intrauterine infection/inflammation-induced lung injury.

**Methods:**

An animal model of intrauterine infection/inflammation-induced lung injury was established with pregnant Sprague–Dawley rats inoculated with *Escherichia coli* suspension. The intrauterine inflammatory status was assessed through the histological examination of the placenta and uterus. A serial of histological examinations of the fetal and neonatal rats lung tissues were performed. The fetal and neonatal rat lung tissues were harvested for next generation sequencing at embryonic day 17 and postnatal day 3, respectively. Differentially expressed mRNAs and lncRNAs were identified by conducting high-throughput sequencing technique. The target genes of identified differentially expressed lncRNAs were analyzed. Homology analyses for important differentially expressed lncRNAs were performed.

**Results:**

The histopathological results showed inflammatory infiltration, impaired alveolar vesicular structure, less alveolar numbers, and thickened alveolar septa in fetal and neonatal rat lung tissues. Transmission electron micrographs revealed inflammatory cellular swelling associated with diffuse alveolar damage and less surfactant-storing lamellar bodies in alveolar epithelial type II cells. As compared with the control group, there were 432 differentially expressed lncRNAs at embryonic day 17 and 125 differentially expressed lncRNAs at postnatal day 3 in the intrauterine infection group. The distribution, expression level, and function of these lncRNAs were shown in the rat genome. LncRNA TCONS_00009865, lncRNA TCONS_00030049, lncRNA TCONS_00081686, lncRNA TCONS_00091647, lncRNA TCONS_00175309, lncRNA TCONS_00255085, lncRNA TCONS_00277162, and lncRNA TCONS_00157962 may play an important role in intrauterine infection/inflammation-induced lung injury. Fifty homologous sequences in Homo sapiens were also identified.

**Conclusions:**

This study provides genome-wide identification of novel lncRNAs which may serve as potential diagnostic biomarkers and therapeutic targets for intrauterine infection/inflammation-induced lung injury.

## Background

As is an important perinatal risk factor, the intrauterine infection can result in intrauterine inflammation which does harm to not only pregnant women but also to fetuses. Intrauterine infection/inflammation results in lung injury and plays an important role in the development of bronchopulmonary dysplasia (BPD). Our previous research indicated that intrauterine infection/inflammation could result in fetal and neonatal lung injury, which was characterized by activation of the NLRP3 inflammasome, up-regulation of inflammatory cytokines, inhibition of surfactant production, interference of lung interstitial development, and differential expression of miRNAs [1]. However, the precise biological mechanisms of intrauterine infection/inflammation on fetal and neonatal lung injury and lung development are poorly known. So far, there have been no reliable biomarkers that could serve for improving intrauterine infection/inflammation-induced lung injury.

Numerous efforts have been made to understand how lung tissues are injured and patterned during development and maintained by cells throughout intrauterine and extrauterine life, and many scientists have traditionally focused on the protein-coding genome [2, 3]. Historically, much of the non-protein-coding portion of the genome has been regarded as ineffective DNA. However, the rapid development of high throughput technologies has dramatically expanded our understanding of the noncoding genome over the last decade. Long noncoding RNAs (lncRNAs) which are operationally defined as non-protein-coding transcripts of greater than 200 nucleotides constitute the vast majority of the non-protein-coding transcriptome [4]. In recent years, lncRNAs are increasingly recognized as important regulatory components in the pathogenesis of respiratory diseases, such as chronic obstructive pulmonary disease (COPD), lung cancer, and asthma [5–8]. Accumulating evidence identifies lncRNAs as powerful regulators of inflammation and disease [9–11]. Differential expression of lncRNAs has been closely linked to inflammatory lung injury [12, 13], but little is known about the role of lncRNAs in the underlying mechanisms of intrauterine infection/inflammation-induced lung injury.

The study on characteristics of lncRNAs expression in developmental phases of lungs with injury after intrauterine infection/inflammation has not been reported yet. Because rats and humans have strong biological similarities, and *Escherichia coli (E. coli)* is a common pathogen of intrauterine infection, we utilized pregnant Sprague–Dawley (SD) rats inoculated with *E. coli* suspension as a model and applied next-generation sequencing (NGS) and bioinformatics techniques to perform genome-wide analyses of differentially expressed lncRNAs and mRNAs between the intrauterine infection and control groups. In this study, we have demonstrated that infection/inflammation results in fetal and neonatal lung injury. We have also identified differentially expressed lncRNAs in rats and homologous sequences in humans that may serve as potential diagnostic biomarkers and therapeutic targets for improving intrauterine infection/inflammation-induced lung injury.

## Methods

### Ethical statement

The study is reported in accordance with the ARRIVE guidelines 2.0 (Animal Research: Reporting of In Vivo Experiments) [14]. The research procedures involving animals were performed at Zhejiang University (Hangzhou, Zhejiang, People’s Republic of China) following review and approval by the Laboratory Animal Welfare and Ethics Committee of Zhejiang University (No. ZJU20220063).

### Laboratory animals

Healthy male SD rats, weighing 270–350 g, and female SD rats, weighing 250–320 g, were purchased from the Experimental Animal Center of Zhejiang Academy of Medical Sciences (Hangzhou, China). The rats were housed at room temperature with free access to diet and water. All animal experiments were carried out in accord with the NIH Directive (The eighth edition of the Guide for the Care and Use of Laboratory Animals, published in 2011). Male and female SD rats were mated in cages at a ratio of 1:2 at 20:00. The smear tests of the female rats’ vaginal secretions were performed, and it was recorded as embryonic day 0.5 (E0.5) if the sperm was detected by microscopy at 8:00 in the next morning. The pregnant rats were randomly divided into two groups (*n* = 12 for each group): the normal control and the intrauterine infection group. At the end of the experiment, all rats were euthanized by cervical dislocation after receiving an intraperitoneal injection of 2% pentobarbital sodium (40 mg/kg) for anaesthetization..

### Establishment of the intrauterine infection/inflammation-induced lung injury rat model

A strain of *E. coli* (*E. coli* ATCC-25922 supplied by the bacteriological laboratory of the National Clinical Research Center for Child Health) was prepared into a suspension agent at a concentration of 1MCF (2.5–3.5 × 10^8^ CFU/mL) prior to use. The date-mated SD rats with pregnancies were injected intraperitoneally with 2% sodium pentobarbital at a dosage of 40 mg/kg body weight to induce anesthesia at embryonic day 15 (E15). They were randomly assigned to receive either normal saline (normal control, *n* = 12) or *E. coli* suspension of 1MCF (intrauterine infection group, *n* = 12) intramuscular injections at a dosage of 0.2 mL on both the left and right sides of the cervix. The injections were guided by a vaginal dilator.

### Efficacy assessment

Successful intramuscular injection into the cervix was confirmed by histological examination of the placenta and uterus. The intrauterine inflammatory status was assessed through the histological examination of the placenta and uterus using a CX-21DIN Microscope (Olympus Corporation, Tokyo, Japan). Pregnant SD rats from each group were anesthetized intraperitoneally with sodium pentobarbital (40 mg/kg), and the fetal rats were surgically delivered at E17, E19, and E21, respectively. The fetal and neonatal rat lung tissues were harvested for HE staining and histopathological analysis at E17, E19, E21, and postnatal day (P) 3, 7, 14, respectively. Four μm-thick sections from neutral buffered formalin-fixed rat lung (right upper lobe) tissues embedded in paraffin blocks were stained with haematoxylin and 0.5% eosin solution. The content of the pathological examination included infiltration of inflammatory cells, changes in vascular morphology, alveolar morphology, and the development of alveolar septa.

### Transmission electron microscope analysis for lung tissues

The lung tissues of neonatal rats were harvested for transmission electron microscope analysis at P3. Six neonatal rats from each group had lungs harvested as previously described. Further lung tissue processing was carried out as biological specimen preparation for transmission electron microscopy. Rat lung tissues were first fixed in a 2.5% glutaraldehyde solution and then in a 1% osmium tetroxide solution. After embedding in resin, the resulting blocks were sectioned, placed onto grids, and then stained with 2% uranyl acetate and 3% lead citrate. Imaging of lung tissues, lamellar bodies, and alveolar epithelial type II (ATII) cells was carried out using a Transmission Electron Microscope JEM-1400Plus (JEOL Ltd, Tokyo, Japan). The impact of the intrauterine infection/inflammation on lamellar bodies of ATII cells in situ within the lung tissue was assessed.

### Library construction for RNA sequencing and sequencing procedures

Total RNA was extracted from lung tissues of E17 and P3 by using miRNeasy Mini Kit (QIAGEN, Germany) in accordance with the manufacturer's instructions. Agarose gel electrophoresis was performed to analyze the integrity of RNA and RNA integrity was also assessed by Agilent 2100 Bioanalyzer (Agilent Technologies, Santa Clara, CA, USA).

After the rRNA was depleted from total RNA, the RNA was cleaved into short fragments of 250-300 bp with the zinc-mediated RNA cleavage method. Random primer hybridization was carried out, and the first strand of cDNA was synthesized. The second strand of cDNA was synthesized from dNTPs (dUTP, dATP, dGTP, and dCTP). The terminals of the purified double-stranded cDNA fragments were modified by Taq polymerase, followed by polyadenylation. The terminals of the polyadenylated cDNA fragments were ligated with the adapters. Ampure XP beads were used to screen and collect cDNAs about 200 bp in length. Degradation of the uracil-containing second strand of cDNAs by uracil specific excision reagent (USER) enzyme is followed by PCR amplification of the first strand of cDNAs, and a highly specific cDNA library was constructed.

Sequencing was performed on the Illumina PE150 Genome Analyser (Illumina Inc., San Diego, USA). Raw data were input into Illumina Casava (software version 1.8) for quality checkups mainly through indexes of sequencing error rate, GC content distribution, and data volume. After filtering and quality checking, clean reads were compared to the genome and transcriptome with Hisat2 (software version 2.1.0). The transcripts obtained from the splicing of each lung sample were assembled by Cufflinks (software version 2.0.2). The transcripts with uncertain chain direction or length less than 200 NT were filtered out, followed by comparing with a known database with Cufflinks (software version 2.0.2) and lncRNAs were obtained through coding potential prediction. The mRNA and lncRNA expression value was determined by the method of fragments per kilobase of transcript sequence per million base pairs sequenced (FPKM).

### Genome-wide identification of differentially expressed mRNAs and lncRNAs

Differentially expressed mRNAs and lncRNAs were identified by comparing normal control with the intrauterine infection group at E17 and P3 after the quantitative analyses of the expression matrix of all samples. The significance analyses of mRNAs and lncRNAs expression differences were carried out. The rat genome assembly mRatBN7.2 was included in the gene annotation run. RefSeq assembly accession GCF_015227675.2 submitted by Wellcome Sanger Institute was used for the analysis.

### Prediction of target genes of the differentially expressed lncRNAs

The target genes of all the identified differentially expressed lncRNAs were predicted by co-location prediction algorithms. The threshold of co-location was set as 100 kb upstream and downstream of lncRNAs.

### Gene Ontology (GO) enrichment analysis

GO enrichment analysis was performed with GOseq (software version 2010) to analyze the main function of putative target genes, which was comprised of three parts: molecular function, biological process, and cellular component.

### Kyoto encyclopedia of genes and genomes (KEGG) pathway analysis [15]

KEGG pathway analysis was performed with KOBAS (software version 2.0) to predict the role of identified differentially expressed mRNAs and lncRNAs in the interaction network in various cell activities.

### Homology analyses of differentially expressed lncRNAs

Sequence similarity searching to identify homologous sequences in human genomes was performed in analyses of differentially expressed lncRNAs by using the Basic Local Alignment Search Tool (BLAST, NCBI). The shared lncRNAs which are differentially expressed at both E17 and P3 were analyzed with the database RefSeq RNA—Homo sapiens using Megablast (Optimize for highly similar sequences). Homology analyses were also applied for the lncRNAs with the highest degree at E17 and P3.

### Statistical analyses

Data for the expression of mRNAs and lncRNAs were normalized and clustered by using Cluster (software version 3.0). *T*-Test was used for measuring the statistical significance of mRNAs and lncRNAs expression differences between the two groups. A significative *P*-value (< 0.05) was used as the significant difference standard.

## Results

### Establishment of the intrauterine infection/inflammation-induced lung injury rat model

**Fig. 1:**
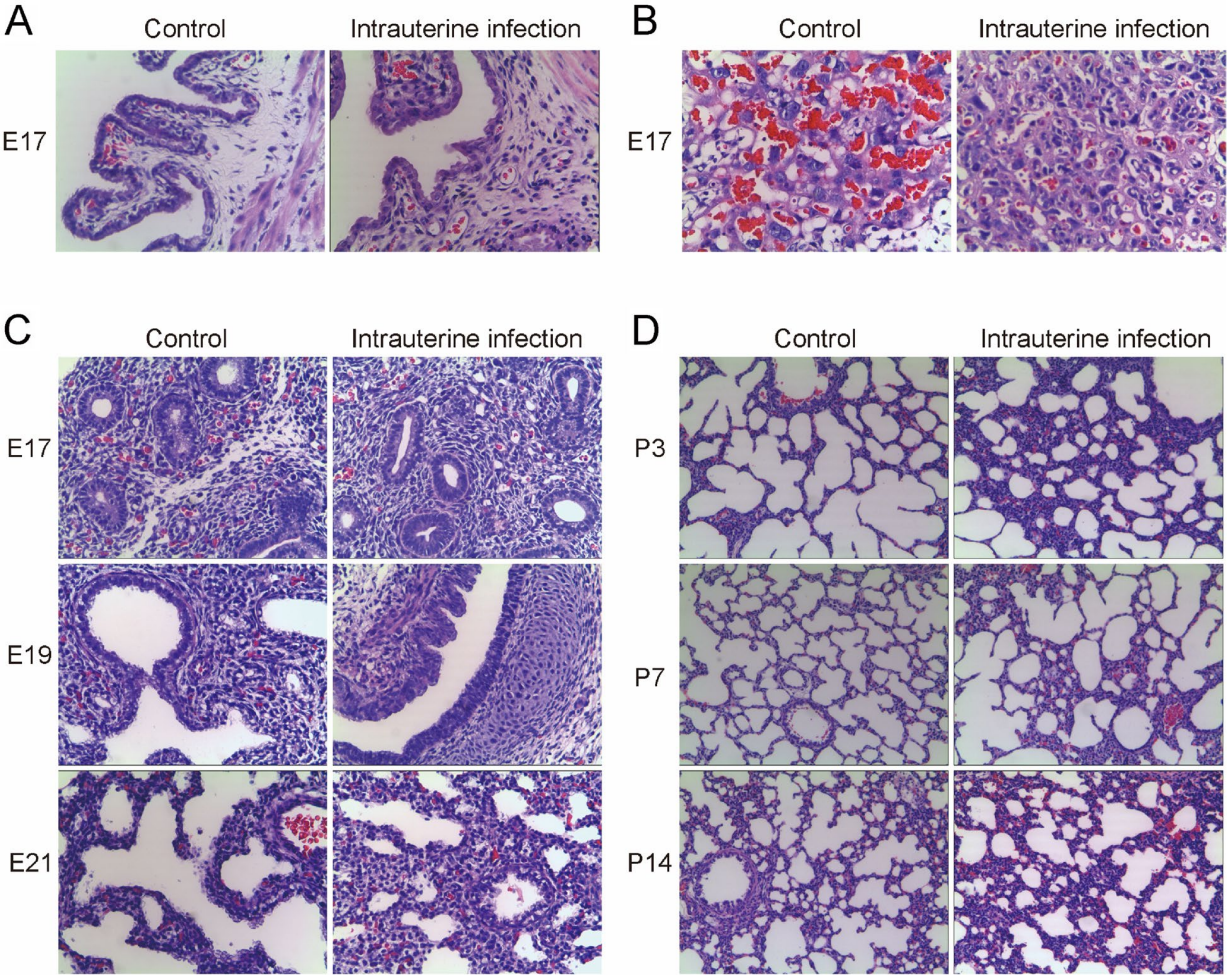
HE Staining for the uterine wall, placenta, and the lungs of fetal and neonatal rats. Legend: HE Staining showed vascular congestion, edema, and neutrophil infiltration of the uterine wall and placenta in the intrauterine infection group at E17. The histological examination showed infiltration, less alveolar numbers, and thickened alveolar septa of the fetal and neonatal lungs in response to intrauterine infection. **A** the uterine wall in the control group and intrauterine infection group (× 200). **B** the placenta in the control group and intrauterine infection group (× 200). **C** the lungs of fetal rats in the control group and intrauterine infection group at E17, E19, E21 (× 200). **D** the lungs of neonatal rats in the control group and intrauterine infection group at P3, P7, P14 (× 100)

Vascular congestion, edema, and marked neutrophil infiltration were found in the placenta and uterine wall of the pregnant rats in the intrauterine infection group compared with the control group (Fig. 1A, B). The histological examination of fetal and neonatal rat lung tissues showed inflammatory infiltration, impaired alveolar vesicular structure, less alveolar numbers, and thickened alveolar septa in the intrauterine infection group compared to the control group (Fig. 1C, D), which were very similar to the pathological changes of lung tissues observed in infants diagnosed with new BPD [1].

### Transmission electron microscopy imaging of lung tissues

**Fig. 2:**
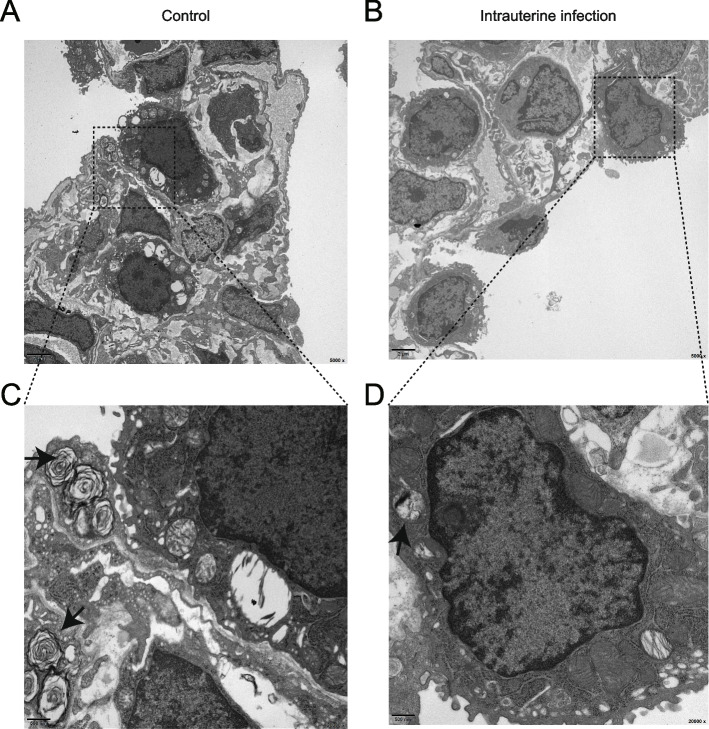
Transmission electron microscopy for the lungs of neonatal rats. Legend: Transmission electron micrographs for the lungs of neonatal rats showed inflammatory cellular swelling, alveolar damage, and less surfactant-storing lamellar bodies in alveolar epithelial type II cells in response to intrauterine infection. (**A**) Control at P3 (× 5000, scale bar 2 μm); (**B**) intrauterine infection group at P3 (× 5000, scale bar 2 μm); (**C**) Control at P3 (× 20000, scale bar 500 nm); (**D**) intrauterine infection group at P3 (× 20000, scale bar 500 nm). Arrowheads mark surfactant-storing lamellar bodies

Transmission electron micrographs revealed detailed ultrastructural alterations in the neonatal rat lung tissues after intrauterine infection/inflammation. The major features in the lungs of the intrauterine infection group comprised inflammatory cellular swelling associated with diffuse alveolar damage. ATII cells were identified by the presence of characteristic lamellar bodies visualized by transmission electron microscopy. Representative images in situ showed inflammatory lung injury and less surfactant-storing lamellar bodies in ATII cells in response to intrauterine infection/inflammation (Fig. 2).

### Genome-wide identification of differentially expressed mRNAs and functional analysis

**Fig. 3:**
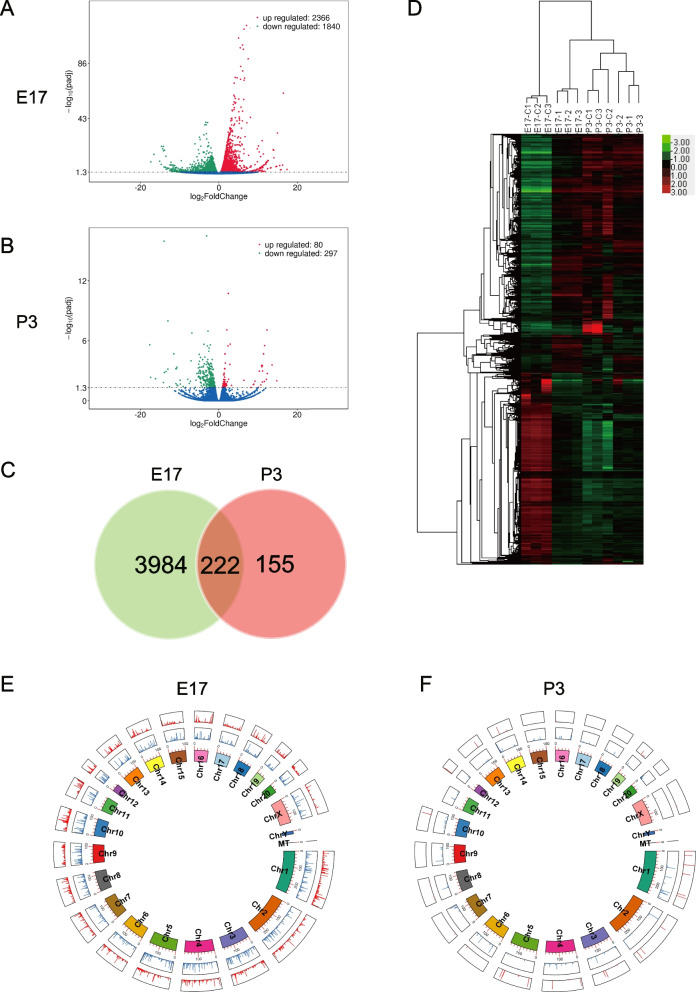
Differentially expressed mRNAs in lung tissues of the intrauterine infection/inflammation-induced lung injury rat model. Legend: (**A**) Volcano map at E17; (**B**) Volcano map at P3; (**C**) Venn diagram for the identified differentially expressed mRNAs at E17 and P3; (**D**) Hierarchical clusterings for the differentially expressed mRNAs, samples with the experimental numbers E17-C1, E17-C2, E17-C3, P3-C1, P3-C2, and P3-C3 were from the control, and samples with the experimental numbers E17-1, E17-2, E17-3, P3-1, P3-2, P3-3 were from the intrauterine infection group; (**E**) A Circos diagram for the differentially expressed mRNAs at E17; (**F**) A Circos diagram for the differentially expressed mRNAs at P3. From the inner to the outer ring, the first ring represents the chromosome map of the rat genome. The second ring represents the distribution of down-regulated mRNAs (blue), and the third ring represents the distribution of up-regulated mRNAs (red)

There were 6 neonatal rat lung tissue samples in the intrauterine infection group (every 3 samples at E17, P3 respectively) and 6 normal neonatal rat lung tissues (every 3 samples at E17, P3 respectively as control) that were profiled by high-throughput dual terminal sequencing. As compared with the control group, there were 4206 differentially expressed mRNAs in the intrauterine infection group at E17, with 2366 up-regulated and 1840 down-regulated (Fig. 3A). There were 377 differentially expressed mRNAs in the intrauterine infection group in comparison to the control group at P3, with 80 up-regulated and 297 down-regulated (Fig. 3B). Both the Venn diagram (Fig. 3C) and hierarchical clusterings (Fig. 3D) of the differentially expressed mRNAs were shown. Circos diagrams for the differentially expressed mRNAs clearly showed the distribution, down-regulation or up-regulation of the mRNAs in the rat genome at E17 and P3 (Fig. 3E,F).

**Fig. 4:**
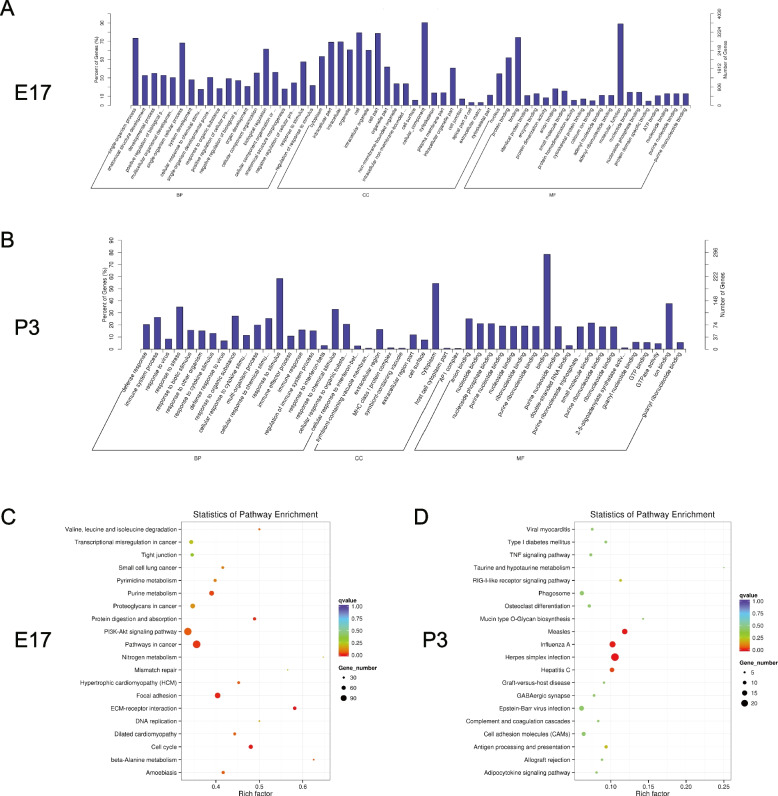
GO enrichment and KEGG pathway enrichment analyses for the differentially expressed mRNAs. Legend: (**A**): GO enrichment analyses for the differentially expressed mRNAs at E17; (**B**) GO enrichment analyses for the differentially expressed mRNAs at P3; (**C**) KEGG pathway enrichment analyses for the differentially expressed mRNAs at E17; (**D**) KEGG pathway enrichment analyses for the differentially expressed mRNAs at P3

The results of GO enrichment analyses revealed that the differentially expressed genes were mainly related to the processes of biological regulation, the cellular component of the intracellular region, and the molecular function of biological binding at E17 (Fig. 4A). They were mainly related to cellular response to different kinds of stimuli, the cellular components of cytoplasm, and the molecular functions of protein binding at P3 (Fig. 4B).

The KEGG pathway enrichment results showed that the identified differentially expressed mRNAs may participate in various developmental processes including extracellular matrix receptor interaction and cell cycle at E17 (Fig. 4C) and they also participate in the processes of infection at P3 (Fig. 4D).

Notably, the expression of Sftpb (surfactant protein B, located in the alveolar lamellar body of ATII cells) was significantly down-regulated at P3, which was consistent with those results of transmission electron microscopy imaging. The expression of Fam20a induced by various cytokines including IL6, IL10, and interferon (IFN)-gamma was significantly up-regulated at E17, whereas it was significantly down-regulated at P3. The expression of Il1rap involved in the positive regulation of cytokine production was significantly up-regulated at P3. These results of differentially expressed mRNAs are consistent with the pathological changes in lung tissues observed in the study.

### Genome-wide identification of differentially expressed lncRNAs in the intrauterine infection/inflammation-induced lung injury

**Fig. 5:**
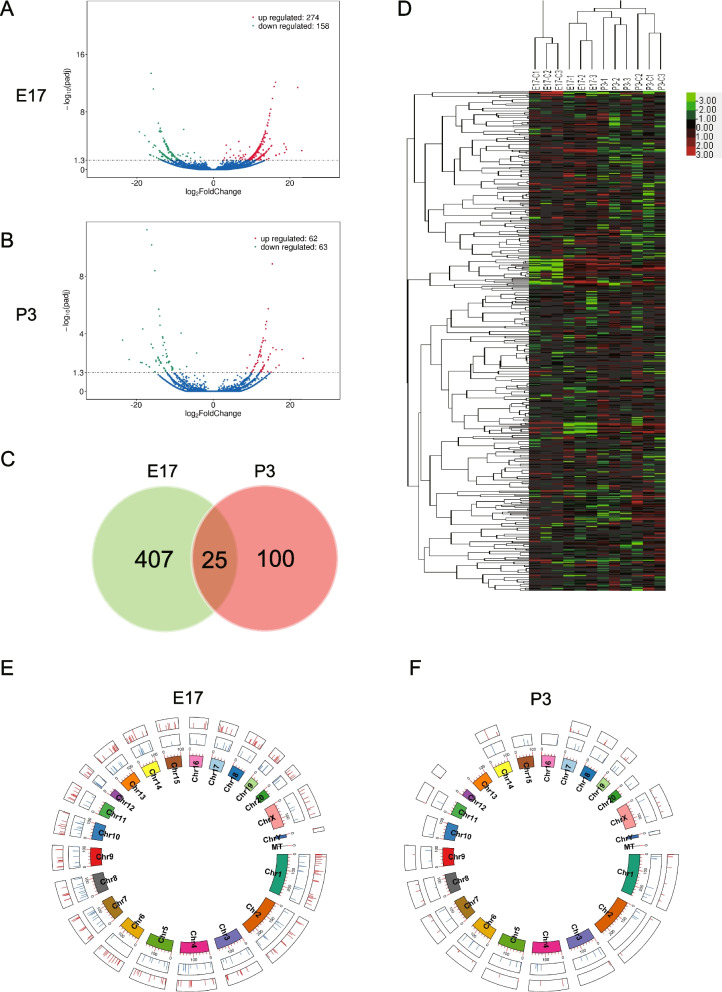
Differentially expressed lncRNAs in lung tissues of the intrauterine infection/inflammation-induced lung injury rat model. (**A**) Volcano map at E17; (**B**) Volcano map at P3; (**C**) Venn diagram for the identified differentially expressed lncRNAs at E17 and P3; (**D**) Hierarchical clusterings for the differentially expressed lncRNAs, samples with the experimental numbers E17-C1, E17-C2, E17-C3, P3-C1, P3-C2, and P3-C3 were from the control, and samples with the experimental numbers E17-1, E17-2, E17-3, P3-1, P3-2, P3-3 were from the intrauterine infection group; (**E**) A Circos diagram for the differentially expressed lncRNAs at E17; (**F**) A Circos diagram for the differentially expressed lncRNAs at P3. From the inner to the outer ring, the first ring represents the chromosome map of the rat genome. The second ring represents the distribution of down-regulated lncRNAs (blue), and the third ring represents the distribution of up-regulated lncRNAs (red)

As compared with the control group, there were 432 differentially expressed lncRNAs in the intrauterine infection group at E17, with 274 up-regulated and 158 down-regulated (Fig. 5A). There were 125 differentially expressed lncRNAs in the intrauterine infection group in comparison to the control group at P3, with 62 up-regulated and 63 down-regulated (Fig. 5B). Logical relations resulting from the two-set Venn diagram show shared lncRNAs that coherently differentiate in the intrauterine infection group at E17 and P3 (Fig. 5C). Hierarchical clusterings of the lncRNAs identified by comparing the intrauterine infection group and control at E17 and P3 were shown in Fig. 5D. Circos diagrams were created for the differentially expressed lncRNAs at E17 and P3, which clearly showed the distribution, down-regulation or up-regulation of the lncRNAs in the rat genome (Fig. 5E,F).

### Target gene prediction and functional analysis of the differentially expressed lncRNAs

**Fig. 6:**
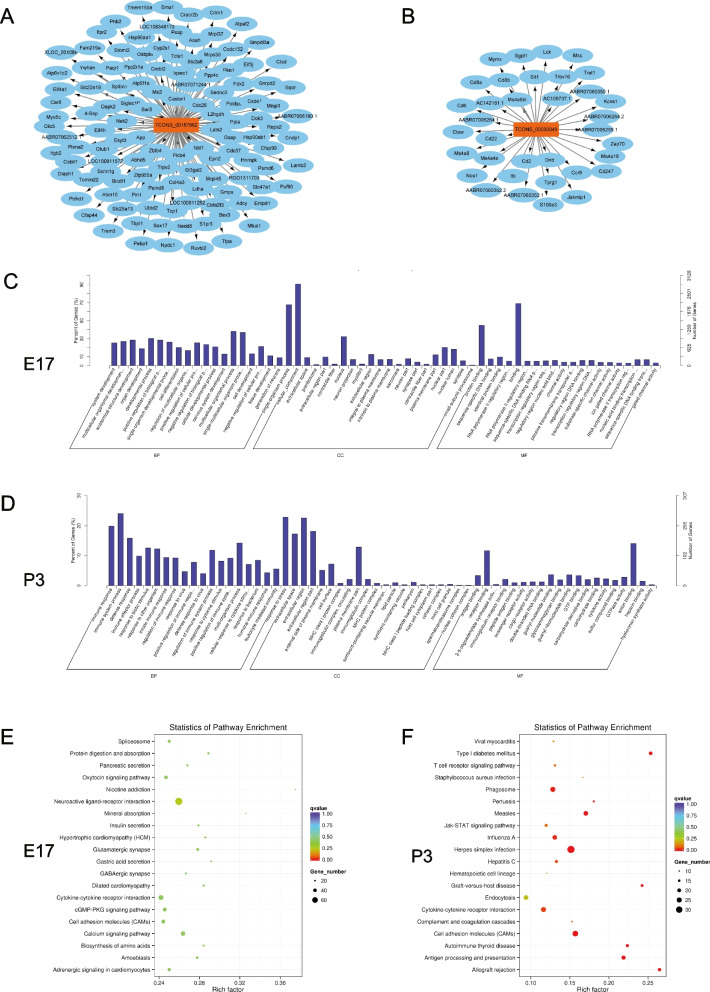
Functional analyses for the differentially expressed lncRNAs. Legend: (**A**) target gene network of lncRNA TCONS_00157962 (a lncRNA with the highest degree at E17); (**B**) target gene network of lncRNA TCONS_00030049 (a lncRNA with the highest degree at P3); (**C**) GO enrichment analyses for the differentially expressed lncRNAs at E17; (**D**) GO enrichment analyses for the differentially expressed lncRNAs at P3; (**E**) KEGG pathway enrichment analyses for the differentially expressed lncRNAs at E17; (**F**) KEGG pathway enrichment analyses for the differentially expressed lncRNAs at P3

Target genes of the differentially expressed lncRNAs in the intrauterine infection group at E17 and P3 were predicted by co-location and co-expression prediction algorithms. There were 3389 target genes of the 432 differentially expressed lncRNAs in the intrauterine infection group at E17, and there were 1443 target genes of the 125 differentially expressed lncRNAs in the intrauterine infection group in comparison to the control group at P3. Based on the results of target gene prediction, the degree of lncRNA TCONS_00157962 (Fig. 6A) was the highest one among the identified differentially expressed lncRNAs at E17, and the degree of lncRNA TCONS_00030049 (Fig. 6B) was much higher than other identified differentially expressed lncRNAs at P3. The degree of lncRNA is defined as the number of other genes that interact with it.

The results of GO enrichment analyses for the differentially expressed lncRNAs showed that the target genes were mainly related to the biological processes of the developmental process, cellular components, and molecular function of protein binding in the intrauterine infection group at E17 (Fig. 6C). The results showed that the target genes were mainly related to the biological processes of the immune system process, response to stress, cellular components of the extracellular region, and molecular function of anion and receptor binding in the intrauterine infection group at P3 (Fig. 6D).

The results of KEGG pathway analysis showed that the identified differentially expressed lncRNAs play an important role in various cell activities including antigen processing and presentation, cytokine-cytokine receptor interaction, and neuro-active ligand-receptor interaction (Fig. 6E, F).

### Homologous sequences in human genomes

**Fig. 7:**
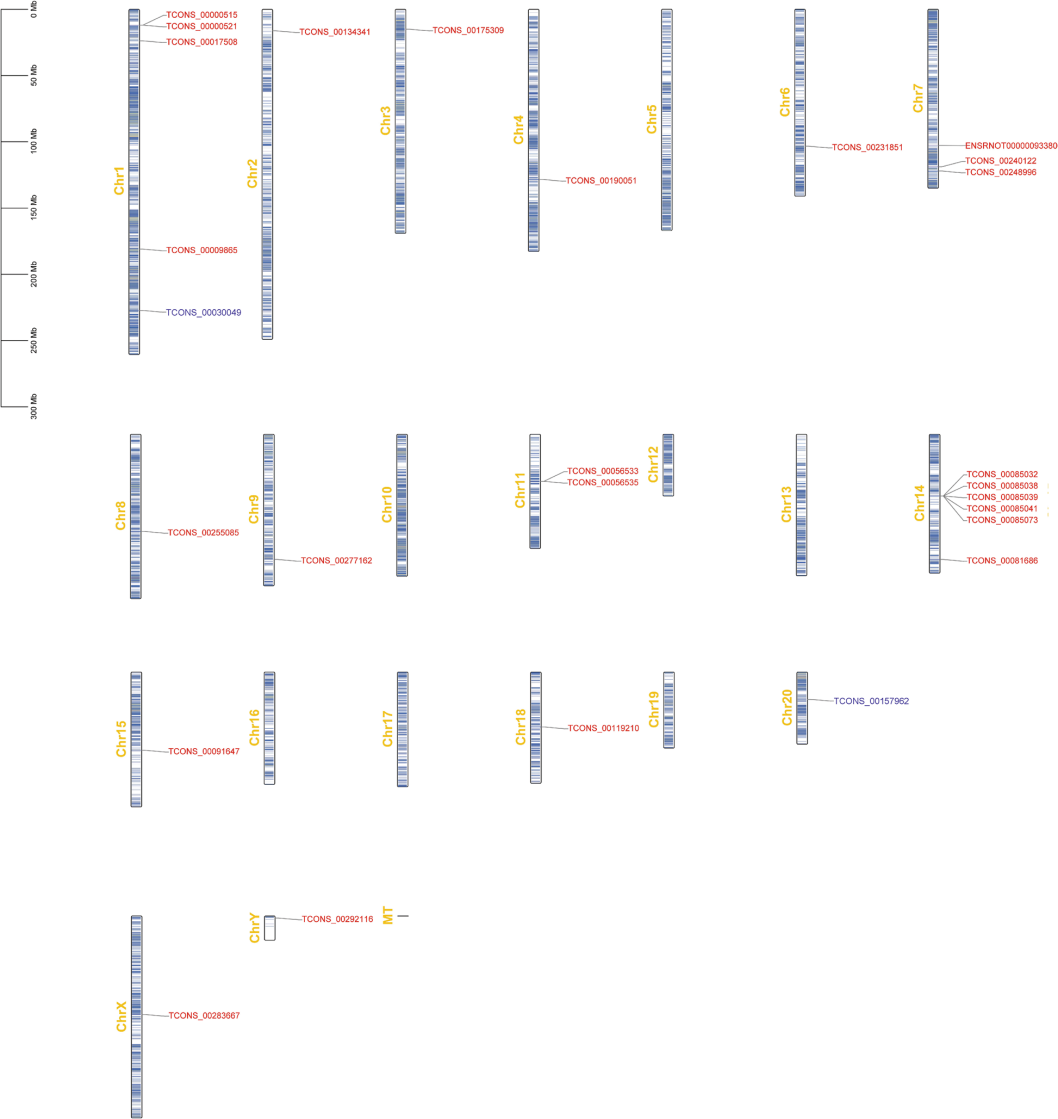
A genetic map for differentially expressed lncRNAs. Legend: Twenty-five shared lncRNAs that were differentially expressed at both E17 and P3 are red. LncRNA TCONS_00157962 (lncRNA with the highest degree at E17) and lncRNA TCONS_00030049 (lncRNA with the highest degree at P3) are blue

**Fig. 8:**
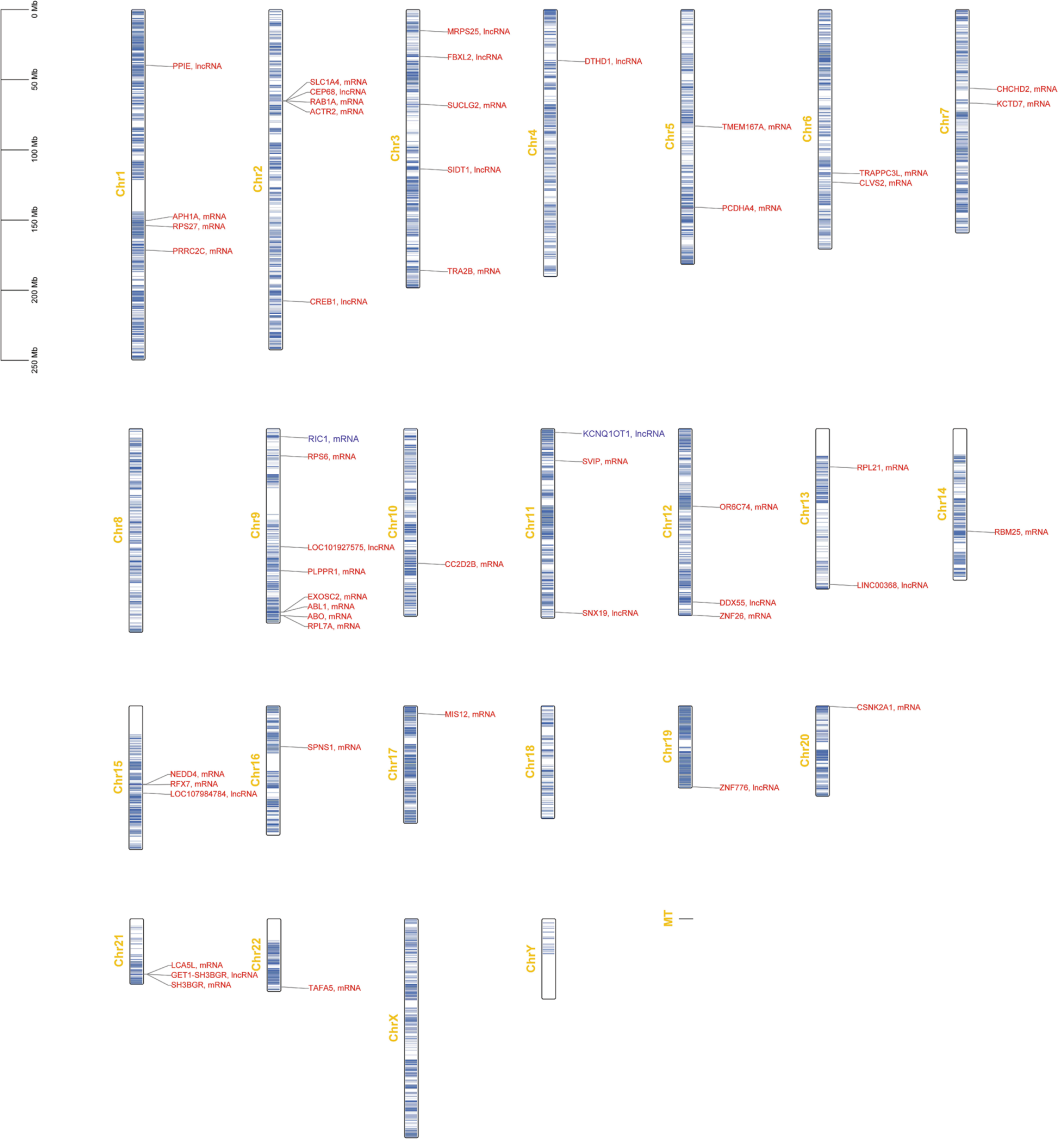
A genetic map for homologous sequences in human genomes. Legend: Homologous sequences corresponding with lncRNAs that were differentially expressed at both E17 and P3 are red. Homologous sequences corresponding with lncRNA TCONS_00030049 (lncRNA with the highest degree at P3) are blue. There was no homologous sequence identified for TCONS_00157962 (lncRNA with the highest degree at E17) in human genomes

Twenty-five shared lncRNAs that were differentially expressed at both E17 and P3 were analyzed by using BLAST. Homology analyses were also applied for lncRNA TCONS_00157962 (lncRNA with the highest degree at E17) and lncRNA TCONS_00030049 (lncRNA with the highest degree at P3). Genetic maps for the 27 differentially expressed lncRNAs in rats (Fig. 7) and their homologous sequences in human genomes (Fig. 8) showed the relative locations of genes and their important features. Detailed information for homologous sequences in Homo sapiens is displayed in Table 1.

**Table 1:** Homology analyses of differentially expressed LncRNAs

LncRNAs in Rattus norvegicus	Homologous sequences in Homo sapiens				
(Transcript locus)	Transcript name	Length (bp)	E value	Identity	NCBI accession number
ENSRNOT00000093380 (chr7:102650147–102744755)	OR6C74, mRNA	9556	1.00E-15	87/105(83%)	NM_001005490.2
TCONS_00000515 (chr1:11908949–11967214)	PRRC2C, mRNA	10381	1.00E-37	120/134(90%)	NM_015172.4
TCONS_00000521 (chr1:11911328–11967214)	PRRC2C, mRNA	10381	1.00E-37	120/134(90%)	NM_015172.4
TCONS_00009865 (chr1:180882154–180949275)	SPNS1, mRNA	1968	2.00E-56	170/193(88%)	NM_001142448.2
	DDX55, lncRNA	2728	1.00E-09	48/51(94%)	NR_135104.2
	PPIE, lncRNA	4292	1.00E-09	48/51(94%)	NR_036544.2
	SNX19, lncRNA	15776	3.00E-05	32/32(100%)	NR_144939.2
	KCTD7, mRNA	4869	3.00E-05	32/32(100%)	NM_153033.5
	MIS12, mRNA	2502	3.00E-05	32/32(100%)	NM_001258217.2
	SIDT1, lncRNA	4392	3.00E-05	32/32(100%)	NR_136285.2
	ZNF776, lncRNA	3187	3.00E-05	32/32(100%)	NR_145326.2
	MRPS25, lncRNA	4237	3.00E-05	32/32(100%)	NR_135246.2
	CSNK2A1, mRNA	12657	3.00E-05	32/32(100%)	NM_177560.3
TCONS_00017508 (chr1:23732451–23778788)	PRRC2C, mRNA	10381	0	1055/1235(85%)	NM_015172.4
	CLVS2, mRNA	11219	4.00E-157	365/395(92%)	NM_001010852.4
TCONS_00030049 (chr1:227157176–227297522)	RIC1, mRNA	6786	1.00E-91	283/328(86%)	NM_020829.4
	KCNQ1OT1, lncRNA	91671	8.00E-04	34/36(94%)	NR_002728.3
TCONS_00056533 (chr11:35464132–35547341)	PRRC2C, mRNA	10381	0	1056/1233(86%)	NM_015172.4
	RPL7A, mRNA	887	1.00E-124	366/425(86%)	NM_000972.3
	SH3BGR, mRNA	1109	6.00E-38	134/154(87%)	NM_007341.3
	LCA5L, mRNA	2808	8.00E-22	98/116(84%)	NM_001384288.1
	GET1-SH3BGR, lncRNA	2131	8.00E-22	76/83(92%)	NR_146618.2
TCONS_00056535 (chr11:35464132–35547666)	PRRC2C, mRNA	10381	0	1056/1233(86%)	NM_015172.4
	RPL7A, mRNA	887	1.00E-124	366/425(86%)	NM_000972.3
	PLPPR1, mRNA	2322	3.00E-80	324/397(82%)	NM_017753.3
	SH3BGR, mRNA	1109	6.00E-38	134/154(87%)	NM_007341.3
	LCA5L, mRNA	2808	8.00E-22	98/116(84%)	NM_001384288.1
	GET1-SH3BGR, lncRNA	2131	8.00E-22	76/83(92%)	NR_146618.2
TCONS_00081686 (chr14:94322933–94598443)	RAB1A, mRNA	2448	0	977/1059(92%)	NM_004161.5
	RPS27, mRNA	352	2.00E-117	284/308(92%)	NM_001030.6
	CEP68, lncRNA	5706	1.00E-54	440/582(76%)	NR_134966.2
	SLC1A4, mRNA	3699	7.00E-37	120/135(89%)	NM_001193493.2
	PCDHA4, mRNA	10526	1.00E-14	85/102(83%)	NM_031500.3
	ACTR2, mRNA	3783	2.00E-13	65/73(89%)	NM_005722.4
	TRAPPC3L, mRNA	2681	9.00E-06	36/37(97%)	NM_001139444.3
TCONS_00085032 (chr14:46517684–46654100)	TRA2B, mRNA	4178	0	1766/2054(86%)	NM_004593.3
	LOC107984784, lncRNA	2353	6.00E-05	34/35(97%)	NR_148210.1
TCONS_00085038 (chr14:46517684–46654102)	TRA2B, mRNA	4178	0	1766/2054(86%)	NM_004593.3
	LOC107984784, lncRNA	2353	6.00E-05	34/35(97%)	NR_148210.1
TCONS_00085039 (chr14:46517684–46654104)	TRA2B, mRNA	4178	0	1766/2054(86%)	NM_004593.3
	LOC107984784, lncRNA	2353	6.00E-05	34/35(97%)	NR_148210.1
TCONS_00085041 (chr14:46517684–46654115)	TRA2B, mRNA	4178	0	1766/2054(86%)	NM_004593.3
	LOC107984784, lncRNA	2353	6.00E-05	34/35(97%)	NR_148210.1
TCONS_00085073 (chr14:46633754–46683418)	DTHD1, lncRNA	6722	7.00E-40	311/415(75%)	NR_165630.1
	ABO, mRNA	6478	3.00E-04	34/36(94%)	NM_020469.3
TCONS_00091647 (chr15:58871309–59029119)	TMEM167A, mRNA	4528	2.00E-40	150/177(85%)	NM_174909.5
	CREB1, lncRNA	8383	1.00E-06	37/38(97%)	NR_163947.1
TCONS_00119210 (chr18:41220183–41493083)	CC2D2B, mRNA	4448	6.00E-08	54/62(87%)	XM_024448000.1
TCONS_00134341 (chr2:16174493–16209879)	CC2D2B, mRNA	4448	9.00E-08	44/48(92%)	XM_024448000.1
TCONS_00157962 (chr20:20528512–20536890)	None				
TCONS_00175309 (chr3:14952246–14984980)	PRRC2C, mRNA	10381	0	1058/1234(86%)	NM_015172.4
	ABL1, mRNA	6719	0	1104/1450(76%)	NM_007313.3
	EXOSC2, mRNA	2012	1.00E-25	84/92(91%)	NM_014285.7
	RPS6, mRNA	1369	2.00E-09	41/42(98%)	NM_001010.3
TCONS_00190051 (chr4:128216095–128464064)	SUCLG2, mRNA	2350	2.00E-37	97/100(97%)	NM_003848.4
	SVIP, mRNA	4479	1.00E-04	38/41(93%)	NM_148893.3
TCONS_00231851 (chr6:103313564–103332197)	RBM25, mRNA	6813	0	661/797(83%)	NM_021239.3
TCONS_00240122 (chr7:118711689–118943142)	TAFA5, mRNA	2604	7.00E-126	600/761(79%)	NM_015381.7
	LOC101927575, lncRNA	2031	5.00E-13	68/78(87%)	NR_110995.1
TCONS_00248996 (chr7:121930621–122136469)	APH1A, mRNA	2068	0	1496/1795(83%)	NM_016022.4
TCONS_00255085 (chr8:73194741–73441446)	RFX7, mRNA	11022	0	3594/4004(90%)	NM_001370561.1
	RPL21, mRNA	566	1.00E-152	450/524(86%)	NM_000982.4
	NEDD4, mRNA	7240	3.00E-80	376/478(79%)	NM_001284338.2
	ZNF26, mRNA	17601	8.00E-06	39/41(95%)	NM_001256280.2
TCONS_00277162 (chr9:94425250–94525143)	CHCHD2, mRNA	797	1.00E-129	420/499(84%)	NM_016139.4
	LINC00368, lncRNA	3455	4.00E-05	34/35(97%)	NR_120415.1
	FBXL2, lncRNA	1943	6.00E-04	30/30(100%)	NR_146129.2
TCONS_00283667 (chrX:74316113–74347926)	None				
TCONS_00292116 (chrY:1517943–1538160)	None				

Literature research has found that transcripts of homologous sequences SNX19 (corresponding with lncRNA TCONS_00009865 in rats), KCNQ1OT1 (corresponding with lncRNA TCONS_00030049 in rats), RAB1A (corresponding with lncRNA TCONS_00081686 in rats), CREB1 (corresponding with lncRNA TCONS_00091647 in rats), ABL1 (corresponding with lncRNA TCONS_00175309 in rats), RPS6 (corresponding with lncRNA TCONS_00175309 in rats), NEDD4 (corresponding with lncRNA TCONS_00255085 in rats), and FBXL2 (corresponding with lncRNA TCONS_00277162 in rats) participate in important processes of lung injury [16–25]. These lncRNAs and their homologous sequences may play an important role in intrauterine infection/inflammation-induced lung injury.

## Discussion

Intrauterine infection/inflammation is frequently associated with preterm birth and correlates with an increased risk of lung injury [26–28]. Although advancements in perinatal medicine such as antenatal steroids, intrapartum antibiotic prophylaxis, exogenous surfactant therapy, and lung protection ventilator strategy have greatly improved survival rates for premature newborns over the past few decades, intrauterine infection remains an important cause of respiratory morbidity and mortality in premature infants, especially in extremely low birth weight (ELBW; < 1000 g) infants. Intrauterine infection triggers a significant lung pro-inflammatory and pro-fibrotic response in the fetal and neonatal period, which has been revealed in our previous study [1]. The results of this study highlight, for the first time, the inflammatory specificity of lncRNA expression, and reflect the regulatory significance of a large number of lncRNAs for the onset of intrauterine infection/inflammation-induced lung injury. The genome wide range of lncRNAs were measured in this study and will assist in clarifying relevant mechanisms of intrauterine infection/inflammation-induced lung injury in future studies.

As a common pathogen of intrauterine infection/inflammation in clinic, *E coli* was selected for proinflammatory substance in the rat model. Intrauterine infection/inflammation was induced by an inoculation of *E. coli* into cervix. This rat model has been proved to be a successful animal model of intrauterine infection/inflammation from different aspects by many studies [1, 29–31]. Due to its low costs, convenient handling, and clinical similarity, it has been applied in preclinical research of lung injury, lung development, brain injury, and brain development. Inflammation in the placenta and uterine wall of the pregnant rats in the intrauterine infection group suggests an animal model of intrauterine infection/inflammation is successfully established, which is consistent with previous research results [1]. Rat lungs are in the pseudoglandular stage at E17 and in the saccular stage at P3 [32]. So E17 and P3 which represent the important stages in the development of lung injury were selected as the optimal observation time points in this study. The histological examinations of fetal and neonatal rat lung tissues were very similar to the pathological changes of lung tissues observed in patients diagnosed with new BPD. Alveolar epithelial type II (ATII) cells have critical secretory and regenerative roles in the alveolus to maintain lung homeostasis. Impairment to ATII cells has been demonstrated to contribute to the development of intrauterine infection/inflammation-induced lung injury through transmission electron microscope analysis in this study. The mRNA expression of *Sftpb* located in the alveolar lamellar body of ATII cells was significantly down-regulated after intrauterine infection/inflammation at P3, which was consistent with those results of transmission electron microscopy imaging. The mRNA expression profile also showed the lungs have abnormal developmental processes after intrauterine infection/inflammation.

As an important class of regulators of gene expression, lncRNAs are comprised of intergenic transcripts, enhancer RNAs, and other sense or antisense transcripts overlapped with genes [33, 34]. LncRNAs have a very wide range of molecular functions, such as gene regulation of allelic expression, organization of nuclear architecture, and regulation of interaction of proteins and RNAs [35]. Great efforts have been made to understand how lncRNAs participate in the pathogenesis of many human diseases [36–39]. So far, some studies have investigated lncRNAs expression in lung injury, but they mainly focused on limited kinds of lncRNAs [40, 41]. The complex genomic research questions in intrauterine infection/inflammation-induced lung injury demand a depth of information beyond the capacity of traditional sequencing technologies. The high-throughput sequencing technology known as NGS has revolutionized our research on lung injury. In this study, the NGS was applied to detect differentially expressed lncRNAs, and there are so many differentially expressed lncRNAs in the process of intrauterine infection/inflammation-induced lung injury as shown in hierarchical clusterings that they may be widely involved in extensive biological processes. Numbers for conjoint (and non-conjoint) differentially expressed lncRNAs as shown in the two-set Venn diagram are also indicated, and some lncRNAs play an important role at different time points. The distribution, down-regulation and up-regulation of the lncRNAs are clearly shown in the Circos diagrams. These results of our study are consistent with a growing number of studies that have indicated that lncRNAs play a crucial role in inflammatory disease progression [42–44].

Although several studies assessed the functions of a few lncRNAs in lung injury [45, 46], the consequence and biological effect of a large number of lncRNAs on lung development remains a challenge. It was shown that differentially expressed lncRNAs could target a wide range of genes. Based on the results of target gene prediction and lncRNA-target gene network analysis, two lncRNAs (lncRNA TCONS_00157962 and lncRNA TCONS_00030049) have been identified as the highest degree lncRNAs. The functions of differentially expressed lncRNAs were inferred and annotated by GO and KEGG pathway enrichment analyses. It was shown that there are dynamic changes in the target gene functions in the development of intrauterine infection/inflammation-induced lung injury. Through regulation of target genes, differentially expressed lncRNAs participate in the developmental process, immune system response process, the molecular function of protein binding process, etc. These processes occur in both cellular components and extracellular regions, which suggests that differentially expressed lncRNAs are widely distributed. The KEGG pathway enrichment results showed that the identified differentially expressed lncRNAs are involved in various important cell activities including antigen processing and presentation, cytokine-cytokine receptor interaction, etc. These important biological activities are crucial in the development of intrauterine infection/inflammation-induced lung injury.

Homology analyses of important differentially expressed lncRNAs have been performed to find out regions of local similarity between rat and human sequences. Some identified homologous sequences have been demonstrated in participating lung injury according to previous studies [16–25]. SNX19 had suggestive single-nucleotide polymorphism association with ammonia-induced acute lung injury and had pathophysiological roles that could be associated with acute lung injury in different ways [16]. Silencing of lncRNA KCNQ1OT1 alleviates lipopolysaccharide-induced lung injury by regulating the miR-370-3p/FOXM1 axis in childhood pneumonia [17]. The RAB1A activity is required for NLRP3 inflammasome activation and inflammatory lung injury. Inhibition of CREB1 which is a transcriptional regulator of Il-6 decreased progranulin-induced intracellular Il-6 production in silica-treated alveolar macrophages cells [47]. Inhibition of ABL1 activity promotes lung regeneration through expansion of an SCGB1A1 + SPC + cell population following bacterial pneumonia [22]. RPS6 activity is closely associated with inflammation, and its expression could be an important biomarker for inflammatory responses [48, 49]. PI3K/AKT/Nedd4-2 pathway is an important pathway in lipopolysaccharide-induced acute lung injury [50]. FBXL2 protein expression attenuates lung injury induced by ischemia–reperfusion [51]. These differentially expressed lncRNAs and their homologous gene families and transcripts may play an important role in intrauterine infection/inflammation-induced lung injury, which warrants further investigation. They seem to have the potential for next-generation prognostic and diagnostic biomarker applications as well as therapeutic targets.

There are several limitations to our study. First, the animal model established with SD rats could not truly simulate the multi-hit theory for lung injury as oxygen toxicity, positive pressure ventilation, invasive procedure, and nutrition deficiency are unfeasible in such a small animal model. Second, the study has not included a cell lineage study because it is difficult to perform the primary culture of respiratory cells in fetal and newborn rats. Third, the study has not explored the long term change of lncRNAs in intrauterine infection/inflammation-induced lung injury and BPD, although our study has made some research on lung development for a relatively long time of 14 days after birth.

## Conclusions

In this study, we conclude that intrauterine infection/inflammation could result in fetal and neonatal lung injury characterized by impairment to alveolar development and damage to ATII cells. Differentially expressed lncRNAs target a wide range of genes and participate in various important cell activities which are crucial in the development of intrauterine infection/inflammation-induced lung injury. Differentially expressed lncRNAs and homologous gene families and transcripts seem to have the potential for prognostic and diagnostic biomarker applications as well as therapeutic targets for improving infection/inflammation-induced lung injury.

## Data Availability

The datasets generated and analyzed during the current study are available in the [OMIX, China National Center for Bioinformation / Beijing Institute of Genomics, Chinese Academy of Sciences] repository, [https://ngdc.cncb.ac.cn/omix/release/OMIX001614].

## Abbreviations

BPD: Bronchopulmonary dysplasia
lncRNAs: Long noncoding RNAs
ATII: Alveolar epithelial type II
GO: Gene ontology
KEGG: Kyoto encyclopedia of genes and genomes
NGS: Next-generation sequencing
*E. coli*: *Escherichia coli*
